# Protective efficacy of bivalent inactivated avian influenza subtype H9N2 and Newcastle disease vaccines commercially available in Bangladesh

**DOI:** 10.5455/javar.2025.l953

**Published:** 2025-09-22

**Authors:** Md. Riabbel Hossain, Shadia Tasnim, Most. Shahana Akter, Munmun Pervin, Jahan Ara Begum, Emdadul Haque Chowdhury, Rokshana Parvin

**Affiliations:** Department of Pathology, Faculty of Veterinary Science, Bangladesh Agricultural University, Mymensingh, Bangladesh

**Keywords:** Newcastle disease virus, H9N2 avian influenza virus, bivalent inactivated vaccine, vaccine efficacy, chicken

## Abstract

**Objective:**

Low pathogenic avian influenza (LPAI) subtype H9N2 and Newcastle disease (ND) are the two major economic diseases worldwide. Continuous genetic evolution of both viruses raises concerns about potential vaccine failure under field conditions. The efficacy of commercially available bivalent inactivated vaccines in preventing ND and avian influenza subtype H9N2 in Bangladesh has not been comprehensively assessed. This study aimed to contribute crucial insights into the evaluated vaccine’s performance against local field strains and to contribute novel data to optimize poultry vaccination strategies in endemic regions.

**Materials and Methods:**

The experimental birds were divided into several groups and were vaccinated according to the manufacturer’s recommendation. Serum samples were collected at regular intervals. Antibody levels against H9N2 and ND virus (NDV) were assessed using hemagglutination inhibition and enzyme-linked immunosorbent assay tests, targeting each virus individually. Following the final booster dose, vaccinated and unvaccinated groups were challenged with locally circulating NDV and H9N2 AI virus strains.

**Results:**

Vaccinated chickens developed robust antibody responses, with titers progressively increasing after each booster and peaking following the final dose. Upon challenge with circulating strains of NDV and H9N2, the immunized birds exhibited no clinical signs of disease. Moreover, no detectable viral shedding of H9N2 was observed, and only minimal NDV shedding was detected in the vaccinated groups.

**Conclusion:**

Our study revealed that all three bivalent inactivated vaccines are effective against LPAI and ND in poultry and elicit a quick and robust antibody response.

## Introduction

Newcastle disease (ND) and avian influenza (AI) are two major infectious diseases of poultry. ND is an extremely contagious and economically significant infectious disease of poultry that is brought on by the ND virus (NDV) [1]. NDV is a negative-sense, non-segmented, enveloped, single-stranded RNA virus that divides into three pathotypes: lentogenic, mesogenic, and velogenic [2]. Since the disease’s first appearance in Newcastle, England, in 1926, outbreaks have spread throughout the world, causing the poultry industry to suffer significant financial losses [2,3]. The poultry industry faces a significant danger from Genotype VII NDV, which is endemic in numerous locations in Asia and Africa, among other genotypes [4–6].

AI is also an economically significant viral disease of poultry caused by the AI virus (AIV), which possesses 16 hemagglutinin (HA) and 9 neuraminidase proteins. The two categories of viruses, according to the intravenous pathogenicity index test, that cause AI are highly pathogenic AIV and low pathogenic AIV (LPAIV) [7–10]. In recent decades, H9N2, an LPAIV with low pathogenicity, has become endemic in poultry in various Asian and African nations, causing significant economic losses for the poultry sector [7,11].

Bangladesh experienced a sharp increase in commercial poultry production in the early 1990s because of improved management techniques, enhanced genetics, and increased market demand [12]. Since 2007, the Bangladeshi poultry industry has grown at a rate of 3%–5% annually, based on FAO production statistics [13]. However, several diseases pose significant obstacles to the rapid progress of this industry in Bangladesh. Infectious diseases alone account for over 30% of chicken deaths, leading to substantial losses in productivity [14]. Along with other infectious diseases, ND and AI have become more frequent in Bangladesh [6,15]. These diseases can manifest alone, in combination with other viruses, or in combination with mycoplasmas and bacteria to form diseases [16]. Among them, low pathogenic AI (LPAI) H9N2 and ND virus have been circulating in Bangladesh for the past two decades, and their continuous genetic evolution has hindered control of the diseases. Therefore, both AI and ND are responsible for huge economic losses in the country [6,17]. In Bangladesh, vaccines for both diseases are available, and the protection rates for H9N2 and ND were reported to be approximately 74% and 60%, respectively, without any clinical signs [18].

According to some earlier studies, both locally produced Australian NDV HR vaccine and conventionally produced locally made BCRDV vaccine provoke humoral immunity that is tested to sustain protection against virulent challenge [19]. In addition, according to studies, a greater immunological response was caused by the LaSota strain compared to the B1 and Clone 30 strains [20]. However, these data are outdated, and no recent information on vaccine protection percentages is currently available. As both NDV and AIV show high genetic diversity, the effectiveness of a vaccine with an old strain may not consistently offer the appropriate amount of protection [21,22]. According to another study, the inactivated ND vaccination of NDV genotype-VII.2, a recent circulating strain, plays an important role in effective control and management of ND [23]; however, this strain is not present in the commercially available vaccines in Bangladesh. To find the most effective vaccinations against circulating strains, it is necessary to continuously assess the protective efficacy of vaccines made from both old and new isolates. This study investigates the efficacy of three commercially available inactivated vaccines that combine NDV and AIV subtype H9N2 in preventing ND and LPAI in layer chickens raised in Bangladesh.

## Materials and Methods

### Ethical approval

The current study has been approved by the ethical committee of the Bangladesh Agricultural University Research System under the approval number BAURES/ESRC/VET/39.

### Investigational vaccines

In Bangladesh, three of the most widely used commercially available bivalent inactivated AI subtype H9N2 and ND vaccines were evaluated for efficacy and safety. The vaccines are imported and marketed by different renowned companies in the country. The code names of vaccines and their compositions are available in Table 1.

### Challenge viruses

For the challenge study, a LPAIV subtype H9N2 of the G1 lineage, previously isolated and stored at the virus repository of the Department of Pathology (A/chicken/Bangladesh/2474-LT86/2021; EPI2187434-41), was used. In parallel, a velogenic strain of NDV, Chicken/Bangladesh/BD-C161/2010 (MK934289-1), belonging to Genotype XIII.2, was selected for the NDV challenge. Importantly, the challenge virus selected for this study belongs to Genotype XIII.2 against the ND and G1 lineages against H9N2, which is currently circulating in the field and associated with recent outbreaks. Therefore, its use is scientifically justified and relevant for evaluating vaccine efficacy against a contemporary and epidemiologically significant strain.

### Experimental design

**Figure 1.**
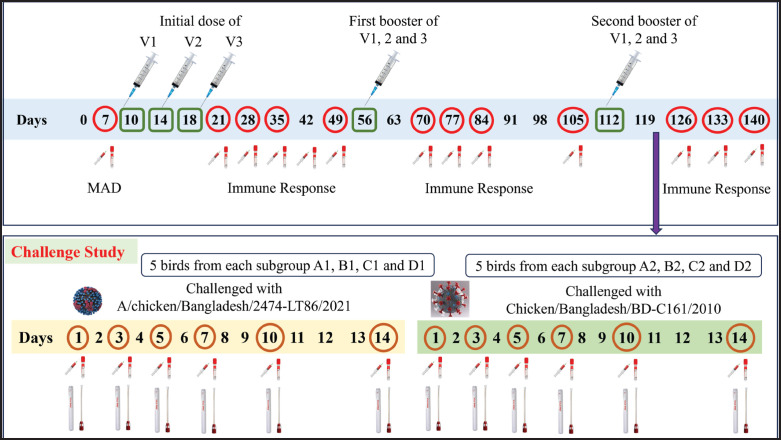
Experimental model of protective efficacy test of bivalent inactivated vaccines. Upper panel showing the vaccination schedule with regular sample collection. Here, red circles indicate the days of serum collection, and green squares indicate the days of vaccination. The lower panel indicated the challenge schedule with the sampling frame. Here, all red circle marks indicate the days of serum and swab sampling.

A total of 200 Shaver Brown day-old chicks of the commercial layer were acquired from Nourish Poultry and Hatchery Limited in Bangladesh. Two hundred chicks were randomly assigned to four groups (A–D), each containing 50 birds. Groups A to C represent three different commercial vaccines (V1, V2, and V3), and group D remained as a negative control (unvaccinated). Each group was further divided into two subgroups—for example, Group A was split into A1 (*n* = 25) and A2 (*n* = 25), and similarly for Groups B (B1–B2), C (C1–C2), and D (D1–D2). The chickens were housed in individual pens under optimal farming conditions for a period of 140 days, with consistent access to feed and appropriate light exposure. Subgroups A1 and A2 received Vaccine 1 (V1), B1 and B2 received Vaccine 2 (V2), and C1 and C2 received Vaccine 3 (V3), while D1 and D2 received Phosphate-buffered saline. The chickens of subgroups A1 and A2 were vaccinated with V1 (0.2 ml) subcutaneously at 18 days; B1 and B2 received a subcutaneous vaccination of V2 (0.2 ml) at 10 days; and C1 and C2 received a subcutaneous vaccination of V3 (0.2 ml) at 14 days according to the manufacturer’s instructions. To measure maternal antibody (MAD), serum samples were taken at random from 10 members of each subgroup at 7 days of age. To check the antibody response post-vaccination, serum samples were taken in the 2nd, 3rd, and 4th weeks following immunization, as well as consecutive weeks of each booster shot (Fig. 1). Serum samples were stored at –20°C before being processed further.

**Table 1.** The vaccine codes and composition.

SL No	The code name of the vaccine	Composition
01	V1	Inactivated AIV A, subtype H9N2 (G1 lineage) and inactivated NDV, strain Ulster 2C (Genotype I class II)
02	V2	Inactivated AIV A, subtype H9N2 (G1 lineage) and inactivated NDV, strain Ulster 2C (Genotype I class II)
03	V3	Inactivated AI type A virus subtype H9N2 (G1 lineage) and NDV, LaSota strain (Genotype II class II)

### Serological assessment

The antibody titer was measured through serum hemagglutination inhibition (HI) titer and enzyme-linked immunosorbent assay (ELISA) separately. The HI was used to assess the antibody titers in each subgroup’s serum sample independently for both H9N2 and ND. The local strains of H9N2 and ND available at the repository were used as antigens for the HI assays, respectively. Additionally, using the ID Screen^®^ Influenza H9 Indirect kit for LPAI and the ID Screen^®^ Newcastle Disease Indirect kit for ND, an ELISA was used to determine antibody titers. The reason for using both HI and ELISA is to evaluate the antibody titer accurately.

### Challenge study

Five birds from each of the subgroups A1, A2, B1, B2, C1, C2, D1, and D2 were placed in different cages for a challenge study after 2 weeks of their final booster. Birds belonging to sub-groups of the corresponding vaccines (A1, B1, C1, and D1) were challenged with the isolate of H9N2, and A2, B2, C2, and D2 were challenged with the isolate of the ND virus. The challenge administered intraocularly and intranasally to the birds was 0.2 ml of a 106.5 50% embryo-infected dose (EID50)/100 μl for H9N2 and 106.4 EID50/100 μl for ND. The HI test was applied to detect the antibody titer at 7, 14, and 21 days post-challenge (dpc).

### Clinical monitoring

The clinical response post-challenge was monitored visually twice daily, and the findings were recorded. Body temperature, feed intake, and feed conversion ratio were measured. The numbers of birds that were healthy, sick, moribund, or dead were noted.

### Viral shedding

To assess viral shedding, oropharyngeal (OP) and cloacal swabs were taken from every 10 birds in the vaccination groups (A, B, and C) and the control group (D) on the 1st, 3rd, 5th, 7th, 10th, and 14th dpc. At 14 dpc, the birds that survived were sacrificed. Samples from the oropharynx and the cloaca were stored at –80°C until further processing.

Viral RNA was extracted using the GeneJETTM Viral DNA and RNA Purification Kit (Thermo Fisher Scientific, USA). A NanoDrop One/OneC Microvolume UV-Vis Spectrophotometer (Thermo Fisher Scientific, USA) was used to test the quality of the RNA, and pure RNA was further used for molecular detection. Real-time quantitative reverse transcriptase polymerase chain reaction (RT-qPCR) was performed targeting the H9 HA gene for LPAI and the M gene for ND using a TaqMan probe-based single-step RT-qPCR assay optimized to detect both the AIV H9 HA and NDV M genes using an AgPath Universal Probe One-Step RT-qPCR Kit (Thermo Fisher Scientific, USA). The final reaction volume was 12.5 μl, containing 2.5 μl of DNA or RNA template, 6 μl of 2× RT-PCR reaction mix, 0.5 μl of RT-PCR Enzyme Mix, 1.5 μl of nuclease-free water, and 2 μl of primer-probe mix (10 pmol each). The RT-qPCR thermocycling conditions were 45°C for 10 min (reverse transcription) and 95°C for 10 min (initial denaturation), followed by 40 cycles at 95°C for 15 sec (denaturation) and 60°C for 1 min (annealing and elongation), with the reading of fluorescence in this step. Samples yielding cycle threshold (CT) values ≤35 were considered positive.

### Statistical analysis

The datasets underwent preliminary evaluation using the Shapiro-Wilk test and box plot analysis to assess the normality of data distribution. Non-parametric tests such as the Mann–Whitney and Kruskal–Wallis tests were used to compare the groups. R 4.4.0 was used in the present study to statistically analyze mean and SE variations. A *t*-test was used to compare the HI and ELISA titers before and after the first and booster shots, and the effectiveness of the vaccine was also checked using one-way analysis of variance in the software Prism 9, with a significance level of *p *< 0.05.

## Results

### Protective efficacy against AIV H9N2 and NDV

Serum samples were collected from both vaccinated and challenged chickens to assess antibody titers following vaccination and post-challenge.

### Immune response post-vaccination

**Figure 2.**
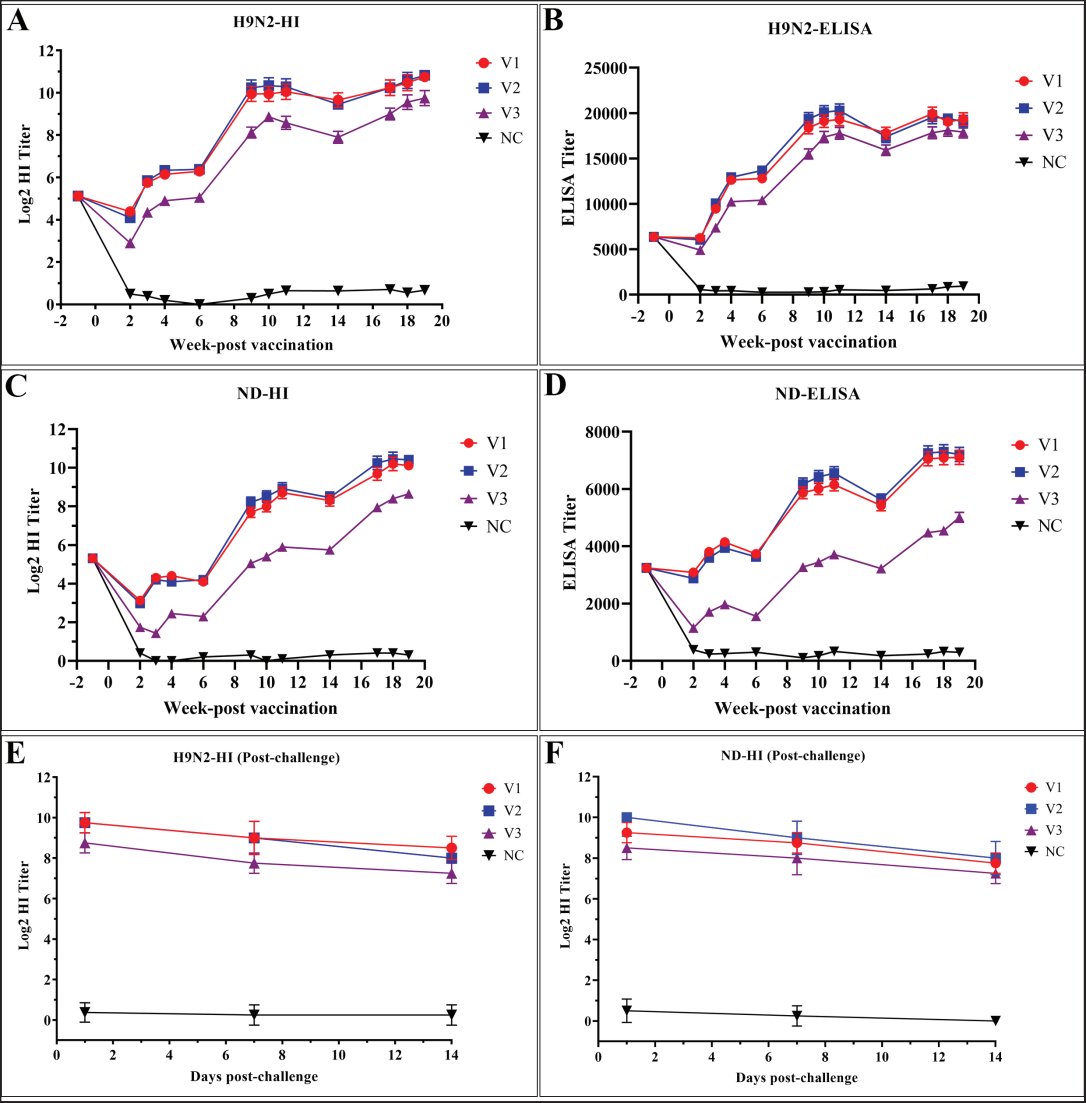
Line graphs showing the antibody dynamics of vaccinated and challenged chickens against AIV H9N2 and NDV. (A) and (B) represent the antibody titers against AIV H9N2 measured by HI (A) and ELISA (B) at different WPV. While (C) and (D) represent the antibody titers against NDV measured by HI (C) and ELISA (D) at different WPV. Following the challenge with local isolates of AIV H9N2 and NDV, the observed HI titer is shown in (E) and (F), respectively.

Following immunization with the bivalent inactivated vaccines (V1, V2, and V3), the HI assay, using the A/chicken/Bangladesh/2474-LT86/2021 H9N2 antigen, showed a mean titer of 5.25 log2 in all vaccinated and unvaccinated (NC) birds during the first week of age, attributed to maternally derived antibodies (MDA). Titers increased following vaccination. At 7 weeks of age [5 weeks post-vaccination (WPV), before the first booster], mean HI titers were 6.45 ± 0.146 for V1, 16.55 ± 0.091 for V2, and 5.10 ± 0.066 for V3 (Table S1). ELISA results showed a similar trend: V1 and V2 had titers of 4.20 ± 0.140 and 4.10 ± 0.133, respectively, while V3 showed a lower titer of 2.25 ± 0.130 at the same time point (Table S2). Peak titers for both HI and ELISA were observed at 18–20 weeks of age (2 to 4 weeks post-second booster) across all three vaccines (Fig. 2A and B).

Similarly, NDV-specific antibody titers increased steadily from two to four WPV. A slight decline was noted before each booster, followed by a rise in titers post-booster. The mean MDA titer for NDV was 5.18 ± 0.162 across all groups. Peak NDV titers were also observed at 18–20 weeks of age, consistent with post-second booster timing (Fig. 2C and D). Notably, V3 elicited a lower antibody response against NDV, especially evident in the ELISA results (Fig. 2D). HI and ELISA titers against NDV at different WPVs are detailed in Tables S3 and S4. No antibody titers were detected in the unvaccinated control (NC) group throughout the post-vaccination period, indicating a lack of protection (Supplemental Tables S1–S4, Fig. 2).

### Immune response post-challenges

To evaluate the efficacy of the inactivated bivalent vaccines against prevalent AIV H9N2 and NDV, selected local strains of each virus were used for the challenge test. Vaccinated chickens had an average HI titer of 10 log2 for AIV H9N2 and 9 log2 for NDV before challenge at 18 weeks of age. Post-challenge, the HI titer remains almost steady with a very negligible decrease up to 14 dpc as shown in Figure 2E for H9N2 and Figure 2F for NDV. In contrast, the unvaccinated (negative control) chickens showed no detectable HI titers against either virus, remained unprotected, and exhibited higher levels of viral shedding along with clinical signs of infection described later.

### Clinical observation post-challenges

**Figure 3.**
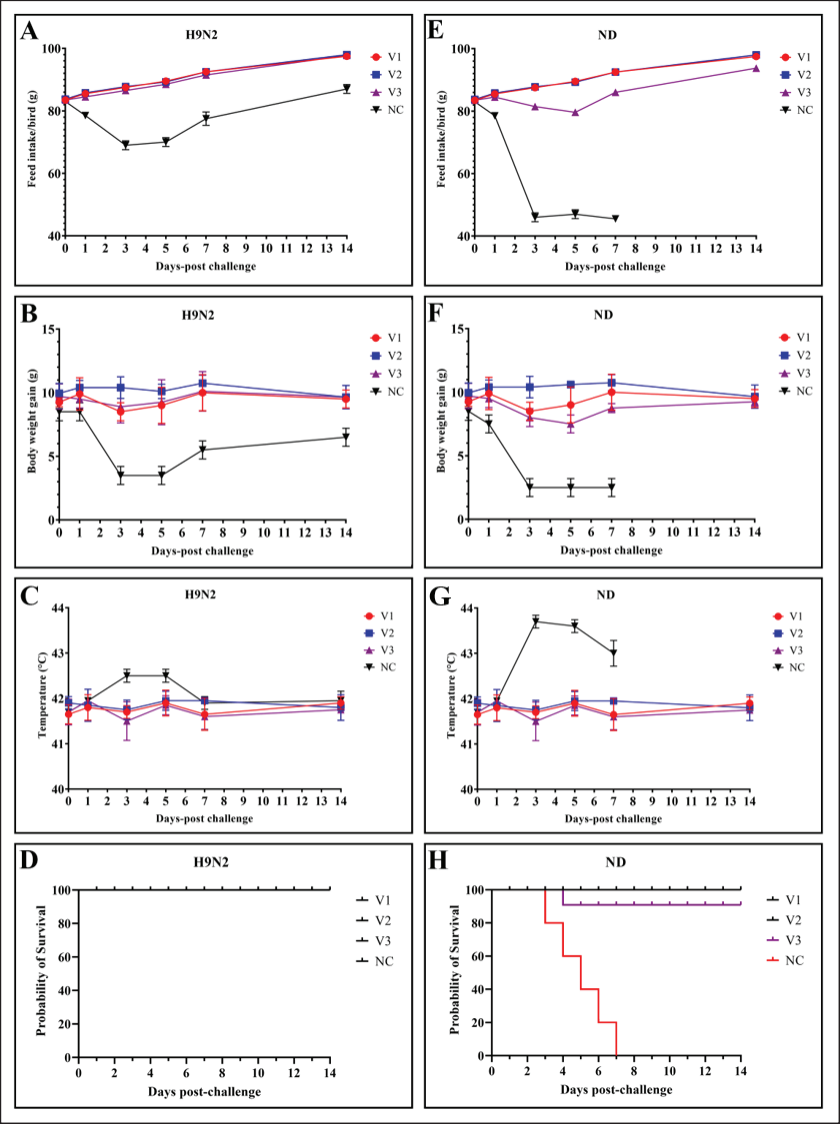
A visual graphic of production parameters and survivability of challenged chickens against LPAIV H9N2 and NDV. The unvaccinated H9N2 challenged group showed insignificant reduction of feed intake (A), body weight gain (B), and insignificant rise of body temperature (C) compared to three vaccinated chicken groups, and survivability was 100% (D), whereas the vaccinated NDV challenged group exhibited a significant reduction of feed intake (E), body weight gain (F), and a significant rise of body temperature (G). Unvaccinated NDV challenged chickens’ survivability was nil within 7 dpc (H).

Clinical parameters such as feed intake, body weight gain, body temperature, and survivability were noted (Fig. 3) for 14 dpc. All vaccinated chickens survived and showed normal feed intake, body weight gain, and body temperature post-challenge. The unvaccinated LPAIV H9N2 challenged chickens showed minor deviation in feed intake and body weight gain: slightly elevated body temperature, conjunctivitis, and ocular discharge with no mortality (Fig. 3A–D). On the other hand, unvaccinated chickens challenged with NDV exhibited typical clinical signs of infection, including depression, anorexia, ruffled feathers, diarrhea, and respiratory distress. A marked reduction in feed intake, impaired body weight gain, and elevated body temperature were observed by 3 dpc, culminating in 100% mortality by 7 dpc (Fig. 3E–H). Except for one death in the V3 vaccine-immunized group (C2) at 4 dpc, the vaccinated chicken groups (A2, B2, and C2) showed no clinical symptoms, visible lesions, or mortality up to 14 days after the challenge.

**Figure 4.**
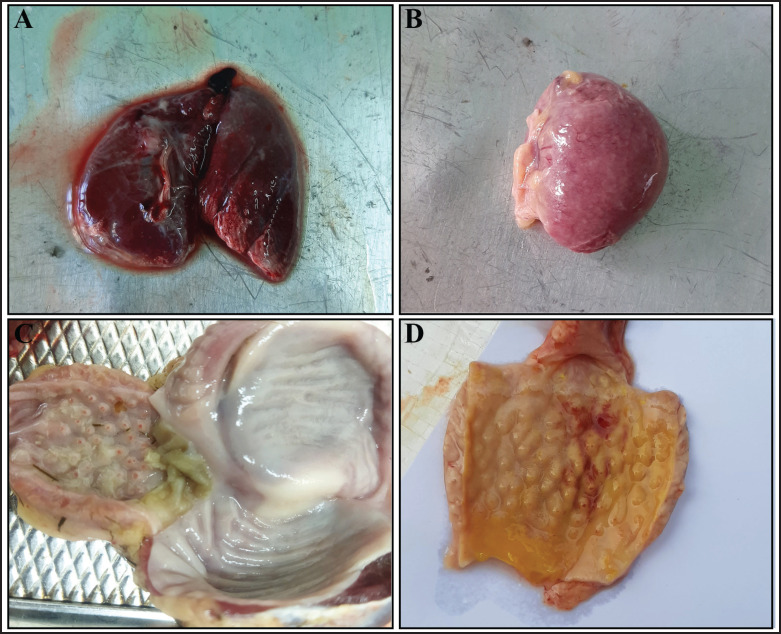
Postmortem changes of unvaccinated chickens challenged with the NDV local strain. The gross lesion indicated congested lung (A), mottled spleen (B), and pinpoint hemorrhage at the proventriculus (C) at 3 dpc, followed by swollen and hemorrhagic proventriculitis at 5 dpc.

Following the NDV challenge, unvaccinated deceased chickens underwent postmortem examination and exhibited severe pulmonary congestion, mottled spleens with white patchy necrosis, and hemorrhages in the proventriculus (Fig. 4A–D), intestines, and cecal tonsils. Interestingly, vaccinated chickens displayed only mild, vaccine-induced hemorrhages localized to the cecal tonsils.

### Shedding of the virus following challenges

**Figure 5.**
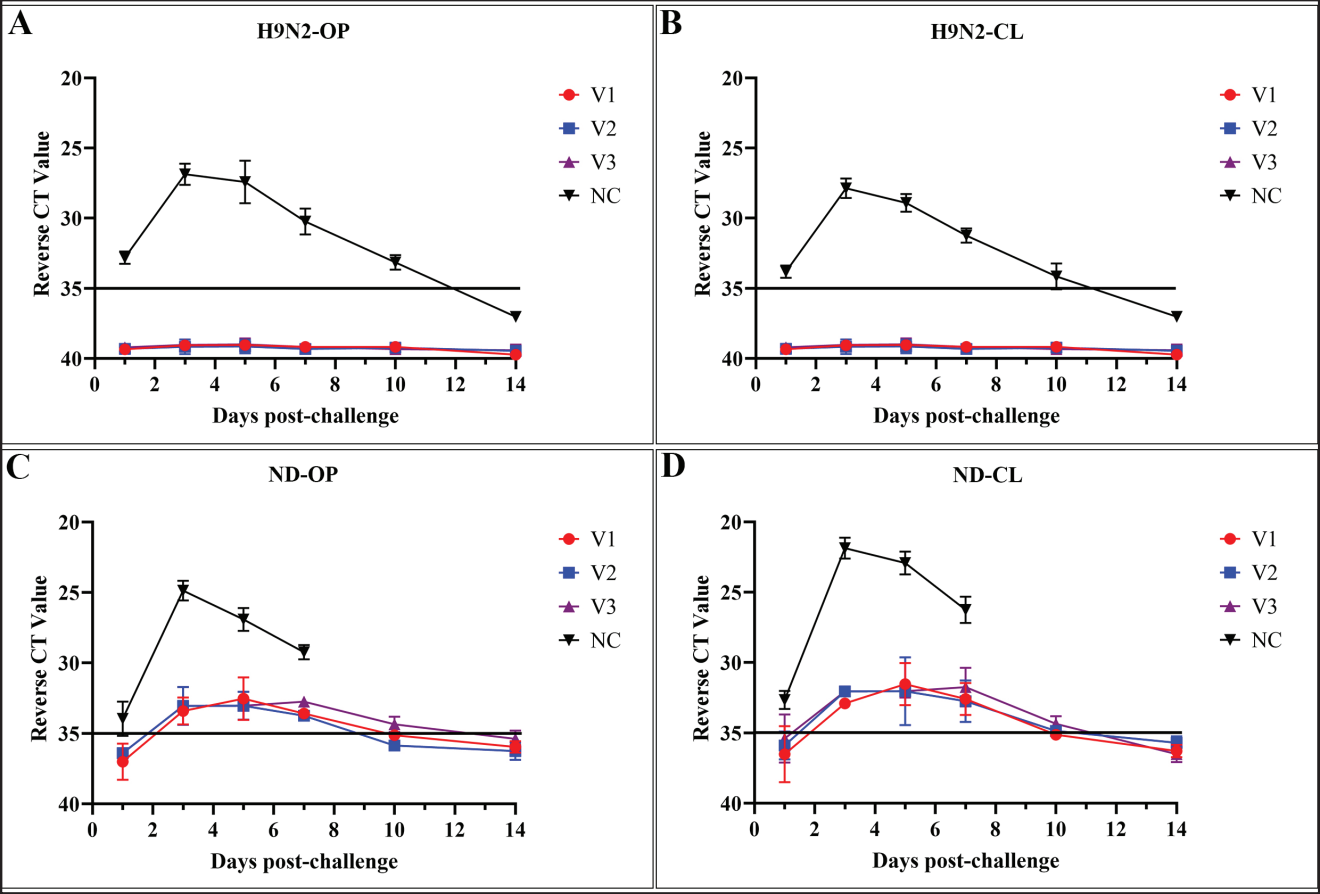
Viral shedding from OP and cloacal (CL) swabs was monitored over 14 dpc. Line graphs illustrate the viral load in OP (A) and CL (B) swabs following H9N2 challenge. Here, V1, V2, and V3 represent the three commercial vaccines, and NC is the negative control that is not vaccinated but challenged with the respective viral strain.

Although no significant clinical signs were observed in the unvaccinated H9N2-challenged chicken group (D1), viral shedding was detected in both OP and cloacal (CL) swabs, with CT values ranging from 31 to 33. In contrast, no viral shedding was detected in the vaccinated subgroups A1, B1, and C1, which corresponded with higher HI antibody titers (Fig. 2E). From the second day post-challenge (dpc) with NDV, all unvaccinated chickens (D2) exhibited typical clinical signs of infection, including depression, ruffled feathers, and diarrhea. A 100% mortality rate was recorded within 7 dpc. All chickens in the NDV-challenged vaccinated subgroups exhibited low levels of viral shedding on days 1, 3, 5, 7, and 10 post-challenge, as determined by RT-qPCR, with Ct values ranging from 34 to 35. In contrast, the unvaccinated group D2 showed significantly higher levels of viral shedding, with Ct values ranging from 24 to 30 (Fig. 5). Additionally, a gradual increase in the HI antibody titer against NDV was observed following the challenge.

## Discussion

This study evaluated the efficacy of the currently marketed and available combined bivalent vaccines of H9N2 and ND to protect currently circulating strains of LPAI and ND within the context of the Bangladeshi poultry production system, particularly under open-shed rearing conditions. LPAIV, H9N2 subtype, was initially identified in 2006, and was extensively spreading throughout the country, producing significant damage to productivity in commercial and backyard poultry [17]. The H9N2 viruses circulating in Bangladesh are descended from the G1 lineage, which originated from China. However, they have evolved significantly through the genetic shift and inter-subtype reassortment with HPAI, H7N3 viruses [24].

Since the first reported case of ND in Bangladesh, the virus has continued to circulate, causing outbreaks and eventually becoming endemic in the country. Among the lentogenic, mesogenic, and velogenic strains, mostly velogenic strains are predominant in Bangladesh, causing severe outbreaks [6]. Currently, Genotype XIII is one of the predominant strains; Genotype VII, particularly sub-Genotype VII.2; and some strains of Genotype II (lentogenic) have been detected in the country [6,25].

Several inactivated and vector-immune vaccines are available in Bangladesh’s market for immunization against H9N2 [26], but challenges of long-term mass vaccination may arise from the viruses’ changes through antigenic drift and inter-subtype reassortment with other subtypes, imposing failure of immunization or no longer protection from current vaccines [27]. Inactivated ND vaccines used in the country are mainly based on the LaSota and the Ulster 2C strains, apart from some experimental genotype-matched vaccines [28] to prevent the disease. The effectiveness of those vaccines may be threatened by the genetic and antigenic diversity of the circulating strains in Bangladesh. The strains such as LaSota (Genotype II) and Ulster (Genotype I) might not offer complete protection against the circulating and virulent strains such as Genotypes XIII and VII [29].

Immunogenicity of AIV and ND is mostly assessed by the HI since it is cost-effective [30]; additionally, the ELISA is another popular choice to evaluate the antibody titer [31]. In this study, both the HI and ELISA antibody titers were considered for the detection of immunogenicity. The findings demonstrated that MAD was present before vaccination, which protects from infection in early life, but not lifelong [32,33]. The present study evaluated the humoral immune response induced by bivalent inactivated vaccines targeting H9N2 AIV and NDV in different vaccination groups. The gradual increase in HI titers observed from two to four WPV reflects a progressive development of specific antibody-mediated immunity following immunization. The vaccine group V1 and V2 (H9N2 with ND Ulster 2C strain) induces an HI titer greater than six log2 against H9N2 infection, and a titer above four log2 against ND is considered the protective threshold [34,35].

Therefore, the antibody responses elicited by all three vaccines can be considered to have reached the protective threshold necessary to prevent infection with both H9N2 and ND viruses. The HI titers remained relatively steady prior to booster vaccination, suggesting a sustained primary immune response. Notably, between 3 and 5 weeks following the first booster dose, group V3 (H9N2 with the Lasota strain of ND) also achieved protective HI titers against ND, indicating a delayed yet effective immune response. Across all vaccine groups, the highest HI and ELISA antibody titers were observed during this period, reaching near-peak levels. These elevated titers were subsequently sustained following administration of the second booster, highlighting the boosting effect of repeated immunization and the importance of a well-structured vaccination schedule to maintain protective immunity over time.

To evaluate the protective efficacy of three selected vaccines, vaccinated chickens along with unvaccinated chickens were challenged with locally isolated LPAIV H9N2 strains of G1 lineage and Genotype XIII.2 (velogenic strains) of NDV. The HI titer after challenge remained similar, with a very negligible decrease observed in both H9N2 and ND. The decrease in antibody titers implies that the vaccines might be working efficiently to prevent viral replication and the elicitation of clinical signs [36]. Challenge results indicated that unvaccinated chickens exposed to LPAIV H9N2 exhibited minor reductions in feed intake and body weight gain, slight increases in body temperature, and mild, transient conjunctivitis, with no observed mortality. Similar clinical signs in unvaccinated, H9N2-challenged chickens were previously reported [37]. All the vaccinated chickens almost did not shed the challenge virus through the OP and cloacal routes, whereas the unvaccinated control shed the virus throughout the 14 days of the challenge observation period. Consistent findings were observed in the case of H9N2, where no viral shedding was detected in vaccinated birds compared to the control group [38].

Unvaccinated NDV-challenged chickens showed the typical clinical signs of infections, such as depression, being off feed, ruffled feathers, diarrhea, and respiratory distress, with a marked reduction of feed intake, body weight gain, and elevated body temperature at 3 dpc, and 100% mortality was noticed by 7 dpc. Postmortem examination of the dead chickens reveals characteristic lesions of ND, like congested and hemorrhagic lungs, petechial hemorrhages in the proventriculus, mottled spleen due to punctate necrosis, and hemorrhages and ulceration in the intestine. The findings were consistent when challenged with velogenic virulent strains of NDV with low HI titer and showed 100% mortality [39,40]. This can be easily aligned with the 100% survival of all the vaccinated chickens of V1 and V2 (Ulster 2C), whereas 90% of V3 (LaSota) survived in the 14-day observation period after the challenge without showing any clinical signs in the case of V3. It is still effective according to the potency assay described by the US Code of Federal Regulations [41]. Challenge study findings reveal that, as opposed to the vaccination, both lentogenic Ulster 2C and LaSota strains are capable of inducing immunity against the heterogeneous velogenic Genotype XIII.2 strain that is currently in circulation within the country [42,43].

All the chickens vaccinated with either Ulster 2C (V1 and V2) or LaSota (V3), along with the H9N2 G1 lineage strain, shed the challenge virus via both OP and cloacal routes, but at much lower levels than in unvaccinated controls. This indicates that the vaccines can only prevent clinical illness, but the vaccinated birds would still shed the virus and may also act as reservoirs for transmission.

## Conclusion

According to the findings of the research, all three inactivated bivalent vaccinations offer protection against common circulating viruses and a good immune response. These vaccines can be employed in the poultry sector of Bangladesh to prevent and control LPAI H9N2 and ND. But compared to the LaSota strain, the Ulster 2C strain consistently elicits an immunological response. The vaccinated birds acted as viral reservoirs and excreted the challenged ND virus despite vaccination.

## List of abbreviations

BCRDV, Baby chick Ranikhet Disease Vaccine; CL, cloacal; CT, cycle threshold; dpc, days post-challenge; EID50, 50% embryo-infected dose; ELISA, enzyme-linked immunosorbent assay; HI, hemagglutination inhibition; LPAI, low pathogenic avian influenza; LPAIV, low-pathogenic avian influenza virus; MAD, maternal antibodies; ND, Newcastle disease; NDV, Newcastle disease virus; OP, oropharyngeal; RT-qPCR, reverse transcriptase quantitative polymerase chain reaction.

## Supplementary Material

**Table S1.** Mean log_2_ HI titer of H9N2 with SEM.

Age (weeks)	Mean ELISA titer of H9N2 ± SEM			
	V1	V2	V3	NC
1	6,625.13 ± 35.78	6,625.13 ± 35.78	6,625.13 ± 35.78	6,625.13 ± 35.78
3	6,405.02 ± 45.09	6,205.02 ± 48.96	5,035.45 ± 39.78	560.69 ± 23.45
4	9,709.39 ± 50.98	10,309.39 ± 53.67	7,568.50 ± 43.78	426.35 ± 21.98
5	12,971.02 ± 54.09	12,271.02 ± 54.89	10,500.66 ± 50.23	437.67 ± 18.90
7	13,126.77 ± 59.98	14,026.77 ± 61.34	10,670.57 ± 56.89	262.77 ± 16.45
10	18,858.54 ± 60.78	19,858.54 ± 62.45	15,898.68 ± 59.09	273.72 ± 14.67
11	19,613.93 ± 65.90	20,613.93 ± 65.89	17,808.50 ± 62.34	327.52 ± 17.90
12	19,796.86 ± 70.24	20,796.86 ± 68.89	18,241.58 ± 64.45	544.83 ± 23.76
15	18,249.09 ± 75.98	17,849.09 ± 79.23	16,319.90 ± 68.67	477.15 ± 25.34
18	20,451.43 ± 74.90	20,051.43 ± 75.78	18,298.54 ± 69.90	633.01 ± 17.89
19	19,017.16 ± 80.98	19,417.16 ± 89.78	18,580.44 ± 73.20	878.42 ± 34.23
20	19,843.13 ± 92.12	19,543.13 ± 94.67	18,375.84 ± 78.98	958.40 ± 42.12

**Table S2.** Mean ELISA titer of H9N2 with SEM.

Age (weeks)	Mean log_2_ HI titer of H9N2 ± SEM			
	V1	V2	V3	NC
1	5.25 ± 0.153	5.25 ± 0.153	5.25 ± 0.153	5.25 ± 0.153
3	4.50 ± 0.126	4.20 ± 0.157	3.00 ± 0.109	0.48 ± 0.103
4	5.90 ± 0.142	6.00 ± 0.109	4.50 ± 0.066	0.38 ± 0.00
5	6.30 ± 0.139	6.50 ± 0.152	5.00 ± 0.133	0.20 ± 0.102
7	6.45 ± 0.146	6.55 ± 0.091	5.10 ± 0.066	0.00 ± 0.000
10	10.20 ± 0.152	10.50 ± 0.122	8.30 ± 0.109	0.30 ± 0.133
11	10.20 ± 0.171	10.60 ± 0.157	8.80 ± 0.133	0.48 ± 0.137
12	10.30 ± 0.119	10.55 ± 0.141	8.80 ± 0.066	0.66 ± 0.131
15	9.90 ± 0.122	9.40± 0.122	8.10 ± 0.141	0.70 ± 0.142
18	10.50 ± 0.154	10.50 ± 0.152	9.20 ± 0.109	0.60 ± 0.141
19	10.75 ± 0.152	10.85 ± 0.157	9.80 ± 0.066	0.60 ± 0.138
20	10.85 ± 0.141	10.95 ± 0.157	10.00 ± 0.109	0.70 ± 0.128

**Table S3.** Mean log2 HI titer of ND with SEM.

Age (weeks)	Mean log_2_ HI titer of ND ± SEM			
	V1	V2	V3	NC
1	5.18 ± 0.162	5.18 ± 0.162	5.18 ± 0.162	5.18 ± 0.162
3	3.05 ± 0.122	2.90 ± 0.056	1.79 ± 0.155	0.40 ± 0.103
4	4.20 ± 0.109	4.10 ± 0.133	1.47 ± 0.067	0.00 ± 0.000
5	4.30 ± 0.122	4.20 ± 0.109	2.50 ± 0.102	0.00 ± 0.000
7	4.20 ± 0.140	4.10 ± 0.133	2.25 ± 0.130	0.20 ± 0.122
10	7.50 ± 0.122	8.00 ± 0.141	5.00 ± 0.122	0.30 ± 0.133
11	7.80 ± 0.066	8.30 ± 0.132	5.50 ± 0.061	0.00 ± 0.000
12	8.50 ± 0.122	8.70 ± 0.061	6.10 ± 0.052	0.10 ± 0.103
15	8.10± 0.133	8.25 ± 0.091	5.80 ± 0.066	0.30 ± 0.142
18	9.45 ± 0.143	10.0 ± 0.141	8.00 ± 0.109	0.40 ± 0.141
19	9.95 ± 0.155	10.2 ± 0.157	8.50 ± 0.141	0.40 ± 0.138
20	10.1 ± 0.141	10.3 ± 0.155	8.60 ± 0.175	0.30 ± 0.128

**Table S4.** Mean ELISA titer of ND with SEM.

Age (weeks)	Mean ELISA titer of ND ± SEM			
	V1	V2	V3	NC
1	3166.93 ± 31.96	3166.93 ± 31.96	3166.93 ± 31.96	3166.93 ± 31.96
3	3012.50 ± 34.09	2812.50 ± 24.82	1122.61 ± 31.56	376.45 ± 12.34
4	3712.98 ± 35.90	3512.98 ± 26.98	1669.45 ± 28.90	228.66 ± 17.90
5	4046.06 ± 42.23	3846.06 ± 29.90	1924.43 ± 28.67	250.40 ± 19.09
7	3642.89 ± 45.09	3542.89 ± 28.09	1521.56 ± 27.90	295.68 ± 13.90
10	5721.99 ± 46.98	6021.99 ± 23.90	3196.37 ± 32.78	101.57 ± 14.98
11	5864.09 ± 35.09	6264.89 ± 35.98	3360.28 ± 36.99	169.44 ± 16.45
12	5998.23 ± 29.09	6398.23 ± 32.91	3645.78 ± 38.45	324.44 ± 14.97
15	5290.81 ± 32.87	6222.00 ± 30.23	3145.36 ± 34.78	177.15 ± 13.78
18	6880.61 ± 36.45	7080.61 ± 35.21	4586.65 ± 32.56	233.01 ± 22.21
19	6917.60 ± 39.02	7117.60 ± 38.02	4657.90 ± 31.45	319.74 ± 21.09
20	6930.48 ± 40.23	7030.48 ± 43.90	5135.98 ± 32.67	292.99 ± 18.79
